# Heterogeneity of Toll-like receptor 9 signaling in B cell malignancies and its potential therapeutic application

**DOI:** 10.1186/s12967-017-1152-5

**Published:** 2017-02-27

**Authors:** Ling Bai, Wei Chen, Jingtao Chen, Wei Li, Lei Zhou, Chao Niu, Wei Han, Jiuwei Cui

**Affiliations:** 1 Cancer Center, The First Bethune Hospital of Jilin University, 71 Xinmin Street, Changchun, 130021 China; 2 ADC Biomedical Research Institute, 1919 University Avenue, Saint Paul, MN 55104 USA; 3 Institute of Translational Medicine, The First Bethune Hospital of Jilin University, 71 Xinmin Street, Changchun, 130021 China

**Keywords:** Cancer immunotherapy, Toll-like receptor 9, B-cell malignancies, CpG oligodeoxynucleotides

## Abstract

Toll-like receptor 9 (TLR9) is expressed in a variety of B-cell malignancies and works as a bridge between innate and adaptive immunity. CpG oligodeoxynucleotides (CpG ODNs), TLR9 agonists, are able to induce anticancer immune responses and exert direct effects against cancer cells, serving as cancer therapeutic agents. Therefore, TLR9 might be a potential therapeutic target for drug development. However, several new evidences have revealed that direct effects of TLR9 agonists on B-cell malignancies is controversial. For example, CpG ODNs can induce apoptosis in certain type of chronic lymphocytic leukemia and lymphoma cells, while induce proliferation in multiple myeloma and other types of lymphoma cells. In this review, we summarize current understanding of the heterogeneity in responses of normal and malignant B cells to TLR9 agonists, due to differences in TLR9 expression levels, genetic alterations (such as MyD88 mutation), and signaling pathway activation. Especially, the downstream molecules of NF-κB signaling pathway play an important role in the heterogeneous response. In order to provide possibilities for therapeutic manipulation of TLR9 agonists in the treatment of these disorders, the preclinical and clinical advances in using CpG ODNs alone and in combination therapies are also summarized in this review.

## Background

B-cell malignancies are clinically and biologically heterogeneous, and they comprise a diverse spectrum of acute or chronic leukemia and lymphoma subtypes. Over the last few decades, advances in chemotherapy regimens, monoclonal antibodies, and targeted therapies have led to a dramatic improvement in the treatment of these disorders [1, 2]. The availability of specific antigens and the easy accessibility of the immune system to these diseases may make the immunotherapy of B-cell malignancies possible. Programmed cell death protein 1 (PD1) antibody and T cells with chimeric antigen receptors (CART) have been considered as promising options for treating B-cell malignancies. However, challenges still remain [3–5]. For example, some drugs are known to suppress the immune system after long-term administration, leaving the patients highly susceptible to infection and relapse [6, 7]. Moreover, the immunotherapy of B-cell malignancies is limited to only some subtypes [8, 9]. Therefore, the identification of novel targets in malignant B cells and the development of low toxicity drugs that can induce anticancer immune responses as well as exert direct effects against cancer cells will provide new strategies for the treatment of B-cell malignancies.

Toll-like receptor 9 (TLR9) plays an important role in the innate immune system and serves as a bridge between innate and adaptive immunity in the antitumor responses [10–13]. Studies have revealed that treatment with CpG oligodeoxynucleotides (CpG ODNs), chemically synthesized TLR9 agonists, leads to tumor regression as a result of T cell- or natural killer (NK) cell-dependent lysis of tumor cells [14]. B-cell malignancies are unique because they express TLR9 and directly respond to CpG ODNs. A recent report has demonstrated that CpG ODNs induce the apoptosis of B-cell chronic lymphocytic leukemia (B-CLL) [15]. Moreover, in contrast to other immune adjuvants, CpG ODNs, administered as an in situ vaccination, have effects on both the immune system and B-cell malignancies [15–18]. Increasing evidence has shown that TLR9 expression on B-cell malignancies exhibits high heterogeneity and that the activation of TLR9 elicits the opposite biological effects [19–21]. For example, CpG ODNs induce apoptosis in some types of human Burkitt lymphoma [22] but enhance the proliferation of malignant B cells in multiple myeloma (MM) [23] and some types of lymphoma [10, 24]. Furthermore, some B-cell lymphomas have no effect during CpG stimulation [25].

The heterogeneity of TLR9 expression on B-cell malignancies and their heterogeneous response to CpG ODNs may provide new targeted therapies against cancerous B cells. The responses of different cancerous B cells to CpG ODNs will determine the appropriate treatment for different B-cell malignancies. However, whether or not TLR9 could serve as a therapeutic target for human B-cell malignancies remains unknown. This review discusses the multiple roles of TLR9 signaling in B-cell malignancies as reported in recent preclinical and clinical studies. Better understanding of the diverse roles of TLR9 signaling will facilitate evaluation of the potential utility of TLR9 as a therapeutic target in the treatment of B-cell malignancies.

### Multiple effects of TLR9-mediated responses

Toll-like receptors (TLRs) are important sensors of foreign microbial components and products of damaged or inflamed self-tissue. The TLRs include at least 10 types of integral membrane glycoproteins in humans that recognize a diverse array of ligands, including bacterial lipopolysaccharides, RNAs, and DNAs. Among these TLRs, TLR9 detects the unmethylated CpG dinucleotides present in viral and prokaryotic genomes, whereas these dinucleotides are generally methylated in host DNA [5, 26]. TLR9 is mainly expressed in the endoplasmic reticulum of human plasmacytoid dendritic cells (pDCs) and B cells, and induces the recruitment of myeloid differentiation antigen 88 (MyD88) to initiate the activation of nuclear factor (NF)-κB, c-Jun N-terminal kinase (JNK), and p38 mitogen-activated protein kinase (MAPK) signaling pathways by binding to its ligand in endocytic vesicles.

**Fig. 1:**
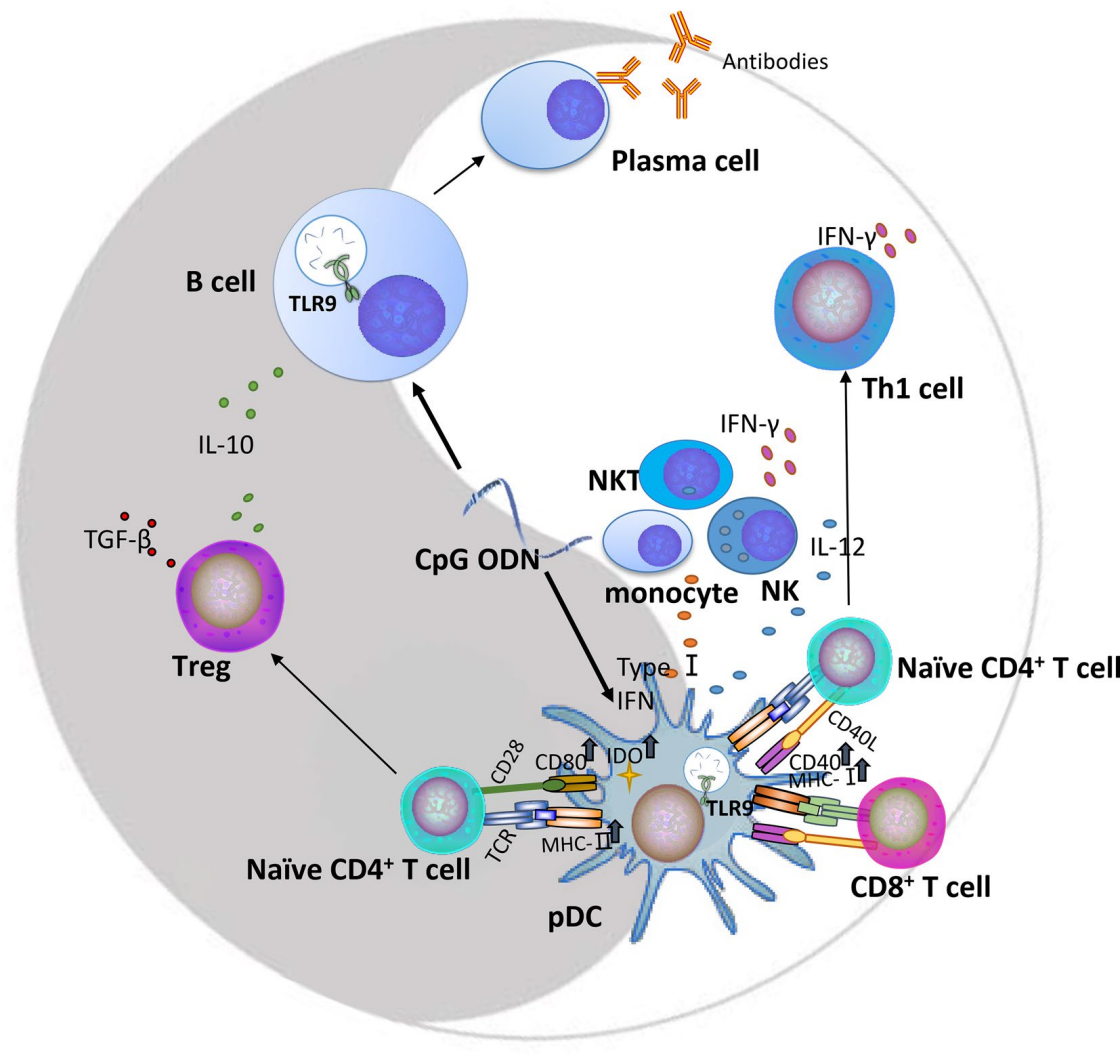
Roles of TLR9 in immune system regulation. CpG ODNs directly stimulate pDCs and B cells. The regulatory effects of TLR9 on the immune system follow a “yin-yang” principle: negative (*dark segment*) and positive (*light segment*) regulation. pDCs maturation is determined by the activation of different signaling pathways, which causes the stimulation of various cytokines and further lead to the activation of different target cells. For instance, CpG ODNs induce pDCs maturation by upregulation of the MHC and costimulatory molecules as well as secretion of cytokines and chemokines, which enhance the capability of stimulating T cells, including promoting T-cell survival and memory, enhancing CD8^+^ T cell cytotoxicity, and activating naive CD4^+^ T cells. Upon TLR9 stimulation, pDCs secrete a large amount of type I IFN, which activates NK cells, NK T cells, and monocytes. Activated NK cells further produce IFN-γ. In addition, matured pDCs subsequently promote Th1 polarization by IL-12 production. On the other hand, matured pDCs increase the expression of indoleamine 2,3-dioxygenase, resulting in the generation of inducible Tregs from naive CD4^+^ T cells with potent suppressor cell function via the secretion of IL-10 and TGF-β. CpG ODNs can also promote B cell proliferation and differentiation into plasma cells. Moreover, TLR9 stimulation in B cells increases the secretion of cytokines such as IL-6 and IL-10

The binding of TLR9 to its ligand also activates interferon regulatory factor-7, resulting in the secretion of type I interferons (IFNs) by pDCs and further promoting pDC maturation [27–29]. Type I IFNs subsequently activate NK cells, natural killer T cells, monocytes, and induce cytotoxic lymphocyte (CTL) and T helper-1 (Th1) responses (Fig. 1). In normal B cells, TLR9 activation increases interleukin (IL) -6 and IL-10 synthesis as well as cell proliferation, immunoglobulin secretion, and plasma cell differentiation (Fig. 1) [30–34]. Although the secretion of IL-10 contributes to B-cell proliferation and “Th1-like” isotype switch, it suppresses the antigen presentation activities and the secretion of Th1-like cytokines (such as IL-12 and IFNs) of pDCs [34–36]. And recent studies indicate that TLR9 can also be expressed on the cell surface of B-cell lymphocytes, serving as a negative regulator of endosomal TLR9 activation [37].

TLR9 stimulation by CpG ODNs appears to induce a strong Th1-type immune response that is therapeutically important for antitumor and antiviral immunities. On the other hand, the increased expression of indoleamine 2,3-dioxygenase in mature pDCs may result in the generation of inducible regulatory T cells (Tregs) from naive T cells. These Tregs play a critical role in maintaining the balance of the immune system [38–41] through secreting IL-10 and transforming growth factor-β.

TLR9 activation also leads to the upregulation of chemokine receptor 7, which causes cell trafficking to the T-cell zone of lymph nodes [42] as well as antigen-presenting molecules (major histocompatibility complex (MHC) class I and II) and costimulatory molecules (CD80, CD86, and CD40), thereby promoting antigen presentation by pDCs and B-cells [11]. Consistent with these findings, a strong synergy has been reported between CD40 ligands and TLR9 agonists in B-cell differentiation [43].

However, the relationship between the TLR9-mediated signaling pathways and cell surface molecules remains unclear. A limited number of experiments have shown that CD19 is a primary costimulatory molecule that amplifies B-cell receptor responses and that CpG ODNs induce CD19 phosphorylation through MyD88/phosphatidylinositol 3-kinase/AKT and Bruton’s tyrosine kinase (BTK) activation in human B cells [44]. Furthermore, BTK inhibitors can block the activation of CD86 and MHC class II by CpG ODNs, suggesting the involvement of BTK in the upregulation of surface molecules in B cells in response to CpG ODNs [45].

In sum, TLR9 mediates both positive and negative immune regulation, which keeps the homeostasis of the immune system [40, 46]. By understanding the deliberate regulation of TLR9 signaling pathways in immune cells as well as malignant B cells, it will help us facilitate manipulating the immune effects of CpG ODNs, such as enhancing antitumor effects in the treatment of cancer and simultaneously avoiding overactive immune response.

### Development of CpG ODN-based drugs as therapeutic agents for B-cell malignancies

Because of multiple immunomodulatory effects, the TLR9 pathway has received increasing attention for the development of cancer therapeutic strategies. TLR9 is stimulated by its ligand-CpG motifs delivered in the form of CpG ODNs that are optimized for their stimulatory activity. These ligands can be chemically synthesized and used as highly selective triggers to stimulate particular subsets of immune cells. Three classes of CpG ODNs have been identified: Class A (Type D), Class B (Type K), and Class C. Class B CpG ODN (CpG-B ODN) is the most efficient in inducing naive B cell (CD19^+^CD27^+^) activation and proliferation [30]. Class C CpG ODN (CpG-C ODN), which exhibits characteristics of both Class A and Class B CpG ODNs, appears to induce more efficient IFN-α secretion than CpG-B ODNs [47–50]. Both CpG-B ODNs and CpG-C ODNs induce a potent Th1-based immune response, leading to comparable antibody production in addition to CD4^+^ and CD8^+^ T-cell responses.

In addition to the high effectivity of CpG-B ODNs for the treatment of B-cell malignancies, CpG-B ODNs (e.g., CpG7909 and GNKG168), as immune adjuvants, have demonstrated excellent safety profiles without dose-limiting, end-organ toxicity, significant laboratory toxicity, and severe adverse effects in over 100 clinical trials. As the development of CpG-based therapy, strategies have been applied to maximize the therapeutic effects of CpG-based drugs, such as prolonging the drug half-life, increasing the cellular uptake and binding, enhancing the drug biological activity, protecting against nuclease degradation [51, 52]. According to the natural capability of certain cells to recognize CpG ODNs, CpG-siRNA/decoyODN conjugates have been harnessed for cell-specific siRNA delivery [53, 54].

Thus, with the development of novel CpG-based agents, it might enhance the direct and indirect effects on cancer cells, which will provide more potential of CpG ODNs in the clinical application.

### TLR9 expression and responses to CpG-B ODNs in normal B-cell subsets

**Fig. 2:**
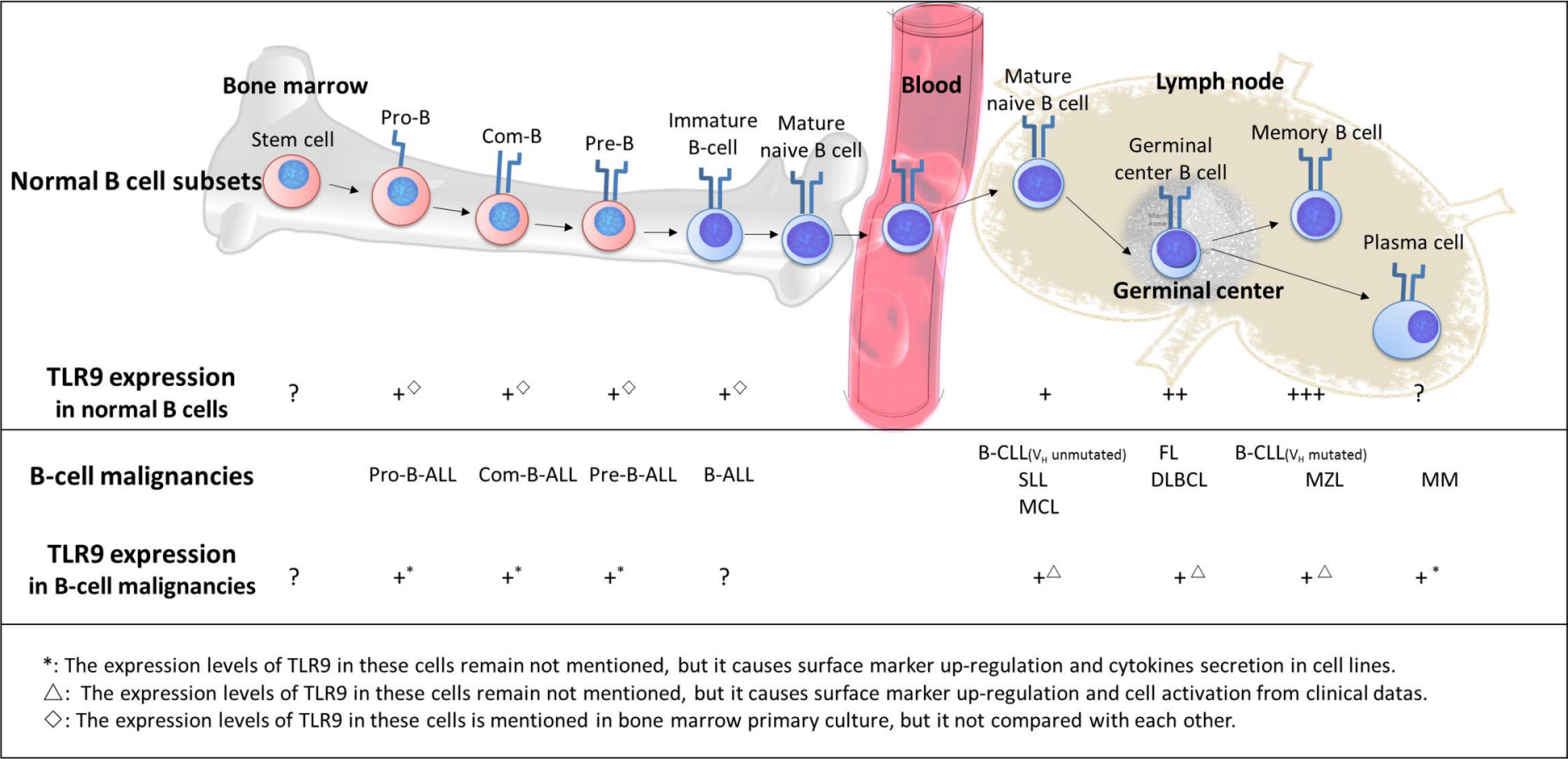
The expression of TLR9 in normal B cells and B-cell malignancies. TLR9 is expressed heterogeneously at almost all stages of B cell development. B cell precursors (+^◊^) grown in *Iscove’s modified Dulbecco’s media* with recombinant IL-7 express different levels of TLR9. Memory B cells express higher levels of TLR9 (+++) than naive B cells (+) and germinal center B cells (++). In addition, B cell malignancies arising from different stages of B cells also express TLR9. Although the exact expression levels of TLR9 in these cells remain unknown, activation of TLR9 causes surface marker upregulation and cytokine secretion according to in vitro experiments (+*) and clinical data (+^Δ^) (*CLL* chronic lymphocytic leukemia, *BL* Burkitt lymphoma, *NHL* non-Hodgkin lymphoma, *MM* multiple myeloma, *GC B cells* germinal center B cells, *FL* follicular lymphoma, *DLBCL* diffuse large B cell lymphoma, *MZLs* marginal zone lymphomas, *MCL* mantle cell lymphoma, *pre-B cells* precursor B cells, *SLL* small lymphocytic lymphoma, *ALL* acute lymphocyte leukemia)

B-cell differentiation, an important step in immune responses against invading pathogens, often involves a germinal center B-cell response, eventually leading to the generation of antibody-producing plasma cells. In addition, other subtypes of B cells, such as peripheral blood-derived naive B cells, germinal center B cells, etc., express different levels of TLR9 [10, 55]. Moreover, higher levels of TLR9 expression are observed in tonsillar B cells than in circulating blood B cells [56]. These findings demonstrate that the differentiation stage has an impact on the expression of TLR9 (Fig. 2). For example, memory B cells express higher levels of TLR9 than naive B cells or germinal center B cells [10]. Some studies suggest that TLR9 expression is related to the level of the B-cell receptor, whose expression is increased with the differentiation of B cells [57]. In addition, a recent study also has shown that newborn naive B cells have a higher TLR9 expression level than adult naive B cells [58]. Although CpG ODNs induce proliferation in human and murine B cells by promoting the cells from G1-phase into S-phase [59, 60], the direct relationship between the response mediated by CpG ODNs and the expression of TLR9 in normal B-cell subsets remains elusive, and needs further study.

### Heterogeneity of TLR9 expression and responses to CpG ODNs in B-cell malignancies

Some B-cell malignancies are characterized by a normal B-cell precursor phenotype, such as CD10 expression. Previous studies have shown that TLR9 is expressed at almost all stages of B cell development [61]. Like normal B cells, B-cell malignancies arising from different stages of B cell development also express TLR9 (Fig. 2). However, recent study indicates that TLR9 expression levels of B-cell malignancies are different from normal B cells [62]. Furthermore, the TLR9 expression in malignant B cells is heterogeneous in each cancer subtype, even in individual patients. Although the heterogeneity of TLR9 expression in some B-cell malignancies remains to be determined, the degree of TLR9-mediated B-cell activation might depend on the expression of TLR9 in B-cell malignancies [63]. However, this is not the case in some instances. For example, despite the same levels of TLR9 expression, memory B cell-related marginal zone lymphomas showed the higher induction of proliferation following stimulation by TLR9 agonists compared to follicular lymphomas and diffuse large B cell lymphomas (DLBCLs) derived from germinal center B cells. Thus, even similar TLR9 expression levels have different responses to TLR9 activation [64].

In addition to the differential expression of TLR9 in normal or cancerous B cells, heterogeneous responses to CpG ODNs also have been observed. Like normal B cells, malignant B cells exhibit heterogeneous responses to CpG ODNs. They can induce either proliferation or apoptosis of different types of cancerous B cells. Clinical data indicate that CpG ODNs can induce the proliferation of marginal zone lymphomas, follicular lymphomas, small lymphocytic lymphomas, diffuse large B cell lymphomas, as well as B-CLL cells from patients with progressive disease and non-mutated V_H_ genes [65]. In contrast, CpG ODNs induce apoptosis of B-CLL cells from patients with stable disease and mutated V_H_ genes. However, CpG ODNs do not have effects on some mantle cell lymphoma cells [63, 66].

**Fig. 3:**
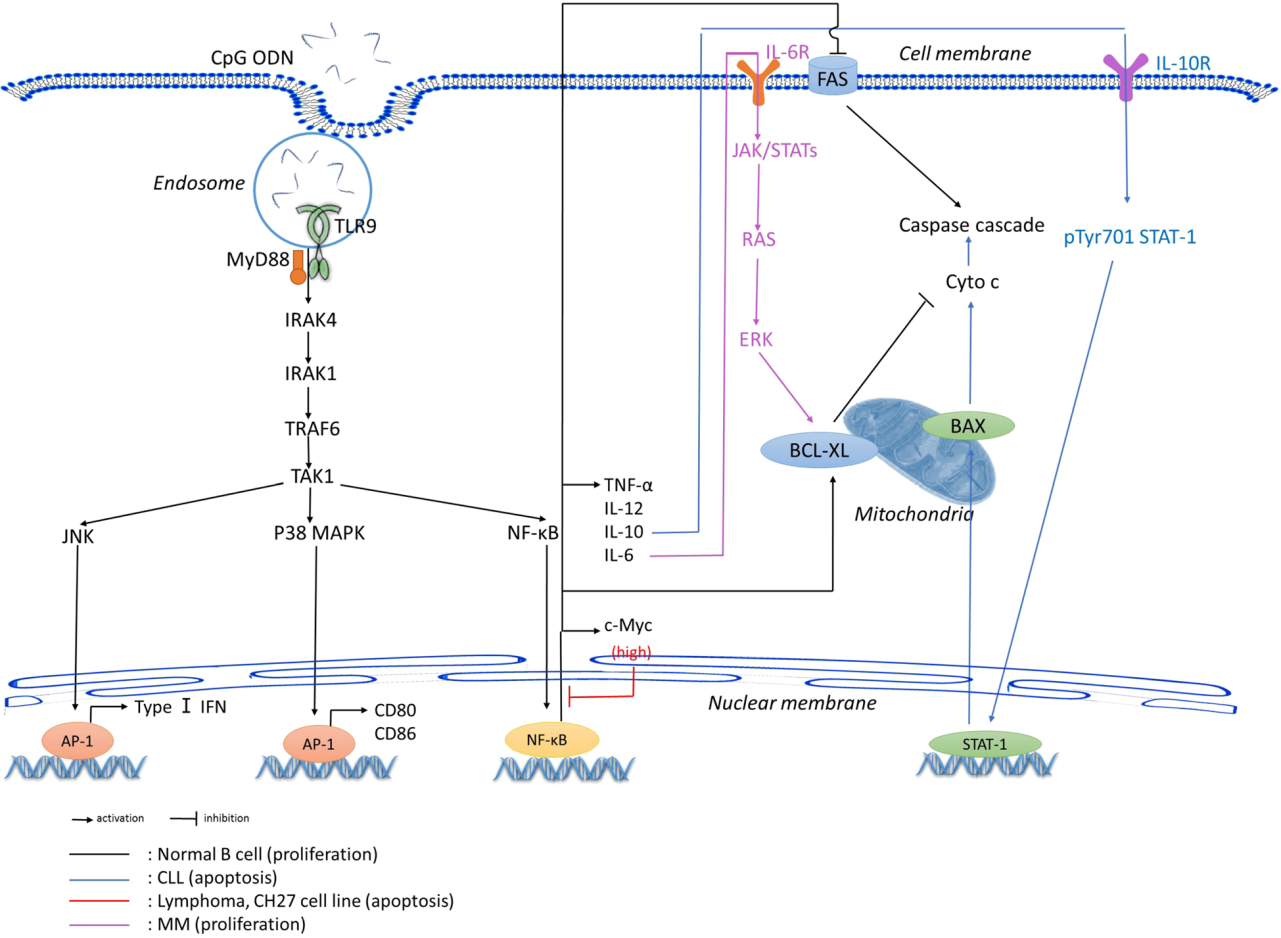
Heterogeneous responses of normal and malignant B cells upon CpG ODNs stimulation. Activation of TLR9 results in the recruitment of MyD88 to initiate the activation of NF-κB, JNK, and p38 MAPK signaling pathways. *Arrows* represent activation. *Bars* represent inhibition. Activation of the NF-κB signaling pathway exerts both antiapoptotic and proapoptotic effects. In normal B cells (*black lines*), activation of NF-κB upregulates the expression of BCL-XL, which blocks cytochrome c release and protects B cells from apoptosis. However, upon treatment of the mouse B-cell lymphoma cell line CH27 with CpG ODNs, overexpression of c-Myc results in transient activation of NF-κB and subsequent inhibition of NF-κB activation (red bar). And then, c-Myc promotes *tumor necrosis factor*-induced apoptosis by decreasing BCL-XL expression and increasing FAS expression. In MM cells (*purple lines*), NF-κB is involved in the activation of Ras-dependent MAPK cascades and the JAK/STAT3 signaling pathway, which is associated with the proliferation of processed IL-6. In B-CLL cells (*blue lines*), activation of the NF-κB signaling pathway induces autocrine IL-10 secretion, which further induces phosphorylation and activation of the signal transducer and activator of transcription 1, resulting in the cleavage and activation of caspases as well as the subsequent apoptosis of B-CLL cells (*MyD88* myeloid differentiation antigen 88, *IFN* interferon, *CLL* chronic lymphocytic leukemia, *MM* multiple myeloma, *MAPK* mitogen-activated protein kinases, *PI3K* phosphatidylinositol 3-kinase, *DR5* death receptor 5, *TRAIL* TNF-related apoptosis-inducing ligand, *STAT1* signal transducer and activator of transcription 1)

Elucidation of the molecular mechanisms underlying the heterogeneity of the TLR9 response may provide a valuable clue for the application of CpG ODNs in the treatment of B-cell malignancies. The TLR9 signaling in malignant B cells mainly involves the NF-κB or MAPK signaling pathway. NF-κB, the major nuclear heterodimer, is expressed and activated in human primary B cells. Its activation exerts both anti-apoptotic and pro-apoptotic effects in response to TLR9 agonists stimulation [67], depending on the downstream molecules of the NF-κB signaling pathway [68]. For instance, if NF-κB is involved in activation of Ras-dependent MAPK cascades and the janus kinase/signal transducers and activators of transcription 3 (JAK/STAT3) signaling pathway, activation will result in the proliferation of IL-6-processed MM [23, 69]. If NF-κB induces phosphorylation and activation of the signal transducer and activator of transcription 1 (STAT1) in B-CLL cells, cleavage and apoptosis are induced via the activation of caspases and poly(ADP-ribose) polymerase [15] (Fig. 3). TLR9 responses to CpG ODNs are also associated with V_H_ gene mutations. The subset of B-CLL samples without a V_H_ gene mutation show strong and durable activation of AKT, MAPK, and NF-κB to CpG ODNs stimulation [65, 70]. Recent research indicates that the apoptosis of B-CLL cells induced by CpG ODNs can be reversed by IL-15- or IL-2-induced Extracellular Signal-Regulated Kinase (ERK) 1/2 and AKT phosphorylation as well as Bcl-2 upregulation [71]. This function may be associated with several chromosomal abnormalities [72]. But in some situation, the gene and protein expression levels may also influence the outcomes of B-cell malignancies upon CpG ODNs treated. For instance, associated with interleukin-10 stimulation, CpG ODNs drive B-cell lymphomas with low c-Myc expression levels proliferation through the activation of both NF-κB and STAT3 signaling pathway and further increasing the expression of the cyclin-dependent kinase 4 [73]. Similarly, another study shows that CpG ODNs trigger sustained increases in NF-κB activation in mouse primary B cells, which also express low levels of c-Myc, thus stimulating the proliferation of primary B cells and the rescue of the B cells from spontaneous apoptosis. However, CpG ODNs could induce cell apoptosis in the cells with overexpression of c-Myc. For example, in the mouse B-cell lymphoma cell line CH27, because of the high expression levels of c-Myc, CpG ODNs trigger a transient NF-κB activation eventually inducing apoptosis via Fas/Fas ligand apoptotic pathway (Fig. 3) [74].

As a key regulator in the TLR9 signaling pathway, the MyD88 mutation is one of the common mutations in B-cell malignancies. Four MyD88 mutations, L265P (Mut1), S219C (Mut2), M232T (Mut4), and S243N (Mut5), have been reported to promote B-cell proliferation. The L265P mutation occurs in 29% of activated B cell-like diffuse large B-cell lymphomas, >90% of Waldenstrom’s macroglobulinemia, and other B-cell lymphoma patients [75]. This mutation causes uncontrolled formation of the protein complex IRAK1/4 and induction of Bruton’s tyrosine kinase signaling, ultimately leading to NF-κB overactivation, elevated levels of STAT3 phosphorylation, IL-6, IL-10, and IFN-β secretion, and subsequent enhanced survival of MyD88 L265P Waldenstrom’s macroglobulinemia cells [75, 76]. A clinical study is currently underway to confirm these findings [77]. In addition, mutations in the TLR/MyD88 pathway, which occur in 4% of CLL patients, can enhance the gene expression of the NF-κB pathway, consistent with the prediction of outcome [78]. But it remains unclear whether MyD88 mutations impact the sensitivity of B-CLL cells to CpG ODNs stimulation. Therefore, further studies are required to determine whether mutations in TLR9 and its downstream signaling pathway are correlated with different cancerous B cells. Even the same type of malignant B cells may respond differently to CpG ODNs stimulation, using different downstream signaling pathways. For instance, Epstein–Barr virus-negative Burkitt lymphoma Akata31 cells exhibit more extensive apoptosis than Epstein–Barr virus-positive Akata cells following CpG 2006 stimulation. This difference may be due to the single nucleotide polymorphisms (rs5743836 and rs352140) within the TLR9 gene in Akata cells [22].

Malignant B cells also show heterogeneous cytokine secretion and costimulatory molecule expression responding to CpG ODNs stimulation. Following CpG ODNs stimulation, the expression levels of CD40, CD54, CD80, CD86, and MHC class I and II are all significantly increased in B-CLL cells [11], whereas only the expression of CD40 is significantly upregulated in acute lymphocyte leukemia cells [21]. However, in mantle cell lymphoma cells, upregulation of CD80, CD86, and MHC class I is not observed. DLBCL cells also show no increase in the expression of MHC class I and II [63].

In summary, different cancerous B cells will respond differently to TLR9 stimulation because of genetic alterations. The heterogeneous responses of B-cell malignancies to TLR9 stimulation remain a challenge for the clinical application of CpG ODN-based therapies. Further investigation is required to examine the expression profiles of TLR9 and the mechanisms underlying the heterogeneous response to TLR9 stimulation in malignant B cells. Characterization of these profiles and pathways may improve the efficacy of CpG ODNs treatment in combination with specific pharmacological inhibitors.

### Potential therapeutic effects of CpG ODNs in B-cell malignancies

Above all, the TLR9 agonists demonstrates direct effects on certain types of malignant cells, besides its stimulating effects on immune response. It has shown therapeutic potential of TLR9 agonists alone and in combination therapies in B-cell malignancies in vitro and in vivo studies.

Several clinical and preclinical trials have demonstrated that CpG ODNs have direct antitumor effects in monotherapy. For example, an in vitro study has demonstrated that CpG 685, a Class B CpG ODN, induces apoptosis of CLL cells via activation of NF-κB [15]. Moreover, a recent phase I study of CpG 7909 (PF-3512676) [31] in patients with early relapsed CLL has revealed that a multiple weekly subcutaneous dose of 0.45 mg/kg body weight is well tolerated, indicating that multi-dose direct effects on B-cell malignancies. Furthermore, it has no toxicity against normal cells and also shows its activating effects on immune system [64, 79].

Some studies have also shown CpG ODNs’ indirect effects on malignant B cells by activating antitumor immune responses. Intravenous administration of CpG 7909 at dose levels 0.01–0.64 mg/kg three times a week was found to elicit an increased NK cell number and induce NK cell activation in patients with refractory non-Hodgkin lymphoma [80]. Additionally, in some types of B-cell malignancy, CpG ODNs have no direct proapoptotic effects on the malignant B cells and only have immune effects in some cases. CpG ODNs have been shown to induce pDCs maturation to secrete IFN-α and IFN-λ, which results in G2-phase arrested in MM cells co-cultured with pDCs. Instead, there is no direct effects on MM cells treated with CpG ODNs alone in vitro [81–84]. Therefore, TLR9 agonists could perform its therapeutic potentiality on B-cell malignancies through the host immune cells and/or direct effects on the tumor cells [17].

In addition to being an efficacious single agent, CpG ODNs show synergetic effects with other therapies such as chemotherapy, radiotherapy, and target therapy. In the studies of combination of CpG ODNs with chemotherapy agent cyclophosphamide (CTX), the combination group showed better outcome in the patients with lymphoma compared with using CpG ODNs or CTX alone. CTX can directly inhibit the tumor growth and give time for the activation of CD8^+^ T cells mediated by CpG ODNs. Moreover, CTX may inhibits the ratio of tumor-infiltrating Treg cells, so that it breaks the homeostasis of the immune system and further enhances the Th1 response. [17] These similar synergetic effects have also been discovered in other types of tumors, with increased survival time [85–88].

Apart from improving the chemotherapeutic agents’ effects, CpG ODNs also improve radiotherapy outcomes of both immunogenic and non-immunogenic with a high complete tumor remission rate [17, 89] by exerting direct effects on human malignant B cells and indirect effects on the immune system with an increase in the number of tumor-reactive memory CD8^+^ T cells, leading to enhanced antitumor immunity [18, 90, 91]. Besides, the treatment was well tolerated. Additionally, CpG ODNs can protect normal B cells from irradiation-induced cell death and enhance macrophages viability even after irradiation. And this protection may be associated with the upregulation of anti-apoptotic molecules, such as Bcl-xS/L and Bcl-2 in these cells [92]. Thus, it indicates that CpG ODNs can improve radioresistance of the normal immune cells.

Furthermore, CpG ODNs can increase the sensitivity of tumor cells to antibody-dependent cellular cytotoxicity (ADCC) lysis, and potentiate cytotoxicity of ADCC effectors. The activation and expansion of Fc receptor-bearing NK cells through cytokines secretion (such as IL-12) mediated by CpG ODNs may increase the efficacy of rituximab. It has been demonstrated that CpG ODNs, when administered in combination with the CD20 antibody, can enhance the efficiency of rituximab in killing Daudi non-Hodgkin lymphoma cell lines. Besides, the upregulation of CD20 expression levels mediated by CpG ODNs might further promotes the killing effects by rituximab [80, 93]. This effect has also been confirmed in murine lymphoma models. CpG ODNs also can be used as adjuvants for anti-idiotype vaccines to increase the efficiency of monoclonal antibody therapy, depending on the TLR9 expression in host immune cells [94, 95]. Furthermore, rituximab plus intratumoral CpG ODNs, but not systemic CpG ODNs, eradicate up to half of 7-day established 38C13-huCD20 tumors, a syngeneic murine B cell lymphoma expressing human CD20. Additionally, brief and extended-duration administration of CpG 7909 in combination with rituximab has been found to be safe in patients with relapsed/refractory non-Hodgkin lymphoma [96].

To increase the cell specific activity, CpG-siRNA/decoyODN conjugates provide a novel therapeutic strategy. For instanse, STAT3 plays an important role in both cancer cells and tumor-associated immune cells to promote cancer progression and survival with upregulation of BCL-XL protein expression. CpG-STAT3siRNA conjugates shows a better effect not only on the direct killing but also on immune-mediated eradication compared with using CpG ODNs alone in hematologic malignancies with upregulating costimulatory and proinflammatory molecules, downregulating PD-L1 molecule, and increasing the ratio of tumor-infiltrating CD8^+^ T cells [53, 54].

In short, despite the fact that the use of CpG ODNs alone or in combination therapies showed a satisfactory treatment effect in B-cell malignancies, CpG ODNs do not have proapoptotic or antitumor effects in some cases [84]. Thus, it suggests that the care should be taken regarding the application of CpG ODNs in B-cell lymphoma treatment. Accordingly, future studies should aim to elucidate the expression profiles of TLR9 in B-cell malignancies, develop other types of CpG ODNs, and design combination therapies with CpG ODNs for the treatment of B-cell malignancies.

## Conclusion and prospects

CpG ODNs exert multiple effects on the immune system in addition to direct effects on B-cell malignancies. Numerous preclinical and clinical trials have demonstrated the safety and efficacy of CpG ODNs in the treatment of several B-cell malignancies, indicating that TLR9 represents a promising target for the treatment of such disorders. However, some studies have reported that CpG ODN-based therapies elicit tumor-promoting effects. Therefore, the heterogeneous expression of TLR9 and differential effects of CpG ODNs in different B-cell malignancies require further investigation. In addition, CpG ODNs act synergistically with other therapies, and potential combination therapeutic strategies for the treatment of B-cell malignancies may thus achieve the desired outcomes. The use of combination therapies and CpG ODNs administration as an in situ vaccination would maximize the clinical benefit. As a result, deepening the understanding of the oncogenic mechanisms in B-cell malignancies will enable matching treatments with the cancer genotype, thus providing further possibilities for the therapeutic manipulation of TLR9 as a target for the precise treatment of B-cell malignancies.

## Abbreviations

TLR9: Toll-like receptor 9
TLRs: Toll-like receptors
mAbs: monoclonal antibodies
CpG ODN: CpG oligodeoxynucleotide
MyD88: myeloid differentiation antigen 88
PD1: programmed cell death protein 1
CART: T cells with chimeric antigen receptors
JNK: c-Jun N-terminal kinase
CTL: cytotoxic lymphocyte
IFN: interferon
IL: interleukin
MHC: major histocompatibility complex
Th1: T helper-1
NK: natural killer
ALL: acute lymphocyte leukemia
CLL: chronic lymphocytic leukemi
MM: multiple myeloma
DLBCLs: diffuse large B cell lymphomas
pDCs: plasmacytoid dendritic cells
CTX: cyclophosphamide
NF: nuclear factor
MAPK: mitogen-activated protein kinases
BTK: Bruton’s tyrosine kinase
JAK: janus kinase/signal transducers
ERK: Extracellular Signal-Regulated Kinase
STAT3: signal transducer and activator of transcription 3
STAT1: signal transducer and activator of transcription 1
ADCC: antibody-dependent cellular cytotoxicity
